# Supplementary material for: Comparative Assessment of Gold and Carbon Nanoparticles as Tags for Lateral Flow Immunoassay of Fenpropathrin in Green Tea
Source: Foods. 2025 Aug 13;14(16):2806. doi: 10.3390/foods14162806 (PMC12385840; doi:10.3390/foods14162806)
Supplement: Supplementary file 1 [file foods-14-02806-s001.zip › foods-3773275-supplementary.pdf]

Supporting Information

Comparative assessment of Gold and Carbon Nanoparticles as tags for Lateral Flow Immunoassay of Fenpropathrin in Green Tea

Chen Chen [a,1], Jingwei Xia [a,1], Jing Wang [a], Hongxing Wei [a], Qianxin Liang [a], Ziye Feng [a], Qingkui Fang[c], Huimei Cai[a,b], Ruyan Hou [b,*], Hongfang Li [a,b] *

[a] Anhui Provincial Key Laboratory of Food Safety Monitoring and Quality Control, Joint Research Center for Food Nutrition and Health of IHM, Animal-Derived Food Safety Innovation Team of Anhui Agricultural University, College of Food and Nutrition, Anhui Agricultural University, Hefei, 230036, China.

[b] National Key Laboratory for Tea Plant Germplasm Innovation and Resource Utilization, Anhui Agri-cultural University, Hefei 230036, China

[c] Key Laboratory of Agri-products Quality and Biosafety (Anhui Agricultural University), Ministry of Education, Hefei, 230036, China.

[1] These authors contributed equally to this work.

* Correspondence: Lihongfang@ahau.edu.cn

Supplementary Material

Reagents and instruments

Figure Captions

Figure S1. Optimization of K_2CO_3 amount.

Figure S2. The optimization of probe addition for AuNPs-based and CNPs-based LFIA.

Figure S3. Purification efficiency of different clean-up materials.

Figure S4. The stability optimization of AuNPs-based and CNPs-based LFIA.

Table Captions

Table S1. Comparison of the proposed LFIA in this study and the reported immunoassays for FPT.

Table S2. Cross-reactivity rate of FPT with main analogs (n=6).

Table S3. Recovery test for FPT using AuNPs-based LFIA and CNPs-based LFIA (n=6).

Table S4. Detection of FPT in real green tea samples using AuNPs-based LFIA, CNPs-based LFIA, and GC-MS/MS (n=6).

Reagents and instruments

Bovine serum albumin (BSA) was obtained from Merck (Merck, Darmstadt, Germany). Goat anti-mouse IgG was purchased from Shanghai Kinbio Tech. Co., Ltd. (Shanghai, China). Fenpropathrin was obtained from Biovet JSC (Peshtera, Bulgaria). Cycloprothrin, bifenthrin, isocarbophos, chlorpyrifos, thiamethoxam, fenitrothion, and carbofuran were purchased from J&K Scientific Ltd. (Beijing, China). Monoclonal antibody and the corresponding coating antigens for FPT were provided by Jiangnan University. Trisodium citrate, tetrachloroauric acid, K_2CO_3, methanol, acetonitrile, polyvinylpolypyrrolidone, polyvinylpyrrolidone, sodium poly(styrene sulfonate), graphitized carbon black, polyethyleneimine, and other chemical reagents were provided by Sinopharm Chemical Reagents Co., Ltd. (Shanghai, China). The green tea sample was purchased from Darunfa Market (Hefei, China) and was verified to be negative by gas chromatography–mass spectrometry.

Nitrocellulose membrane (Millipore 135) was obtained from Millipore AG (Millipore AG, Zug, Switzerland). A 96-well microplate was purchased from Yunpeng Technology Development Co., Ltd. (Xiamen, China). Sample pad, polyvinyl chloride plates, and absorbent paper were purchased from Shanghai Kinbio Tech. Co., Ltd. (Shanghai, China). A Milli-Q Ultrapure water meter was obtained from Millipore (Massachusetts, USA). The ultraviolet-visible spectrum was carried out by BioTek Instruments, Inc. (Winooski, VT). The transmission electron microscopy image was recorded using a Tecnai G2 F20 microscope (Boynton Beach, FL, USA). Dynamic light scattering (DLS) measurements were performed on a DelsaMaxPro device of Beckman-Coulter (Brea, CA, USA). The three-dimensional XYZ dispensing platform and automatic programmable strip cutter were obtained from Kinbio Tech Co., Ltd. (Shanghai, China).

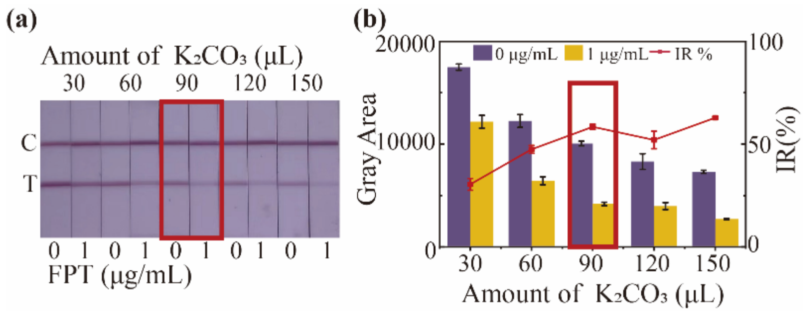

Figure S1. Optimization of K_2CO_3 amount. The (a) visual image of test strips, and (b) the corresponding color intensity of the T line and inhibition ratio for AuNPs-based LFIA. Each test was repeated three times (n=3). The color intensity was recorded with the gray area.

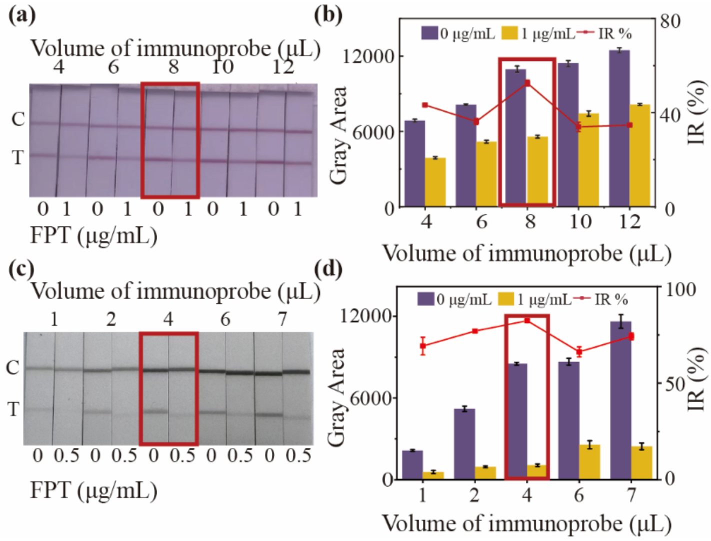

Figure S2. The optimization of probe addition for AuNPs-based and CNPs-based LFIA. Images of the test strip with the addition of different volumes of probe for (a) AuNPs-based and (b) CNPs-based LFIA. The corresponding color intensity of the T line and inhibition ratio for (c) AuNPs-based and (d) CNPs-based LFIA. Each test was repeated three times (n=3).

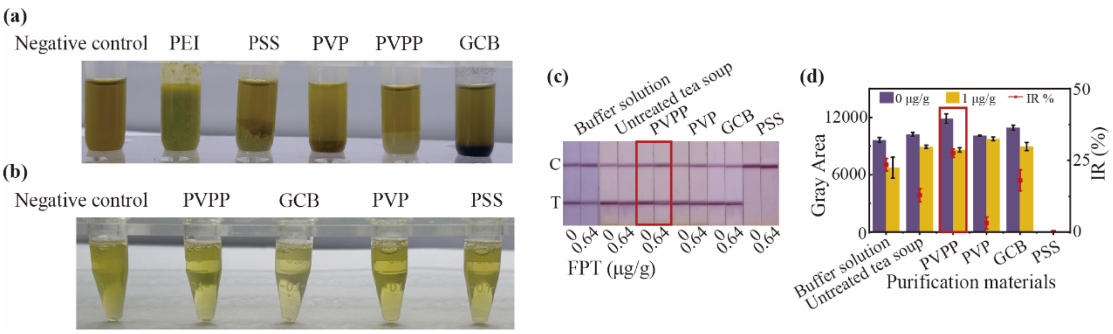

Figure S3. Purification efficiency of different clean-up materials. (a) Images of the green tea extract solution following the addition of the purification reagent and (b) subsequent centrifugation. (c) Images of the test strip after applying the purified green tea extract solution, together with the (d) measured T line color intensity and the corresponding inhibition ratio.

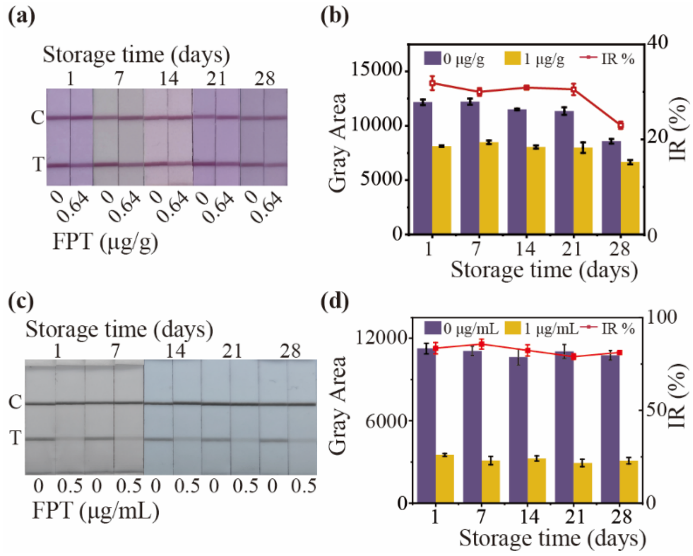

Figure S4. The stability optimization of AuNPs-based and CNPs-based LFIA. (a) Images of the test strip, (b) color intensity of T line and the corresponding inhibition ratio with the storage time from 1 to 28 days for AuNPs-based LFIA. (c) Images of the test strip, (d) color intensity of T line and the corresponding inhibition ratio with the storage time from 1 to 28 days for CNPs-based LFIA. Each test was repeated three times (n=3).

Table S1. Comparison of the proposed LFIA in this study and the reported immunoassays for FPT.

Detection method	Analyte	Sample	qLOD	vLOD	Detection time	Ref.
Fluorescence Immunoassay	Fenpropathrin	Cabbage, Lettuce, Pear, and Apple	0.012 µg/g	-	20 min	1
Fluorescence immunoassay	Fenpropathrin	wheat and maize	-	1.56 µg/kg	15 min	2
Fluorescence immunoassay	Fenpropathrin and Procymidone	Tomatoes, cucumbers, and cabbage	0.114 and 0.082 µg/kg	8.15 and 7.98µg/kg	60 min	3
LFIA	Fenpropathrin, Chlorpyrifos, Imidacloprid, Thiamethoxam, Acetamiprid, Carbendazim, Chlorothalonil, Pyraclostrobin, and Iprodione	Tea	-	5, 2, 0.5, 2, 0.05, 0.5, 0.5-1, 2, and 2 µg/g	30 min	4
LFIA	Fenpropathrin	Apple and Cucumber	62±6 µg/L	-	10 min	5
AuNPs-LFIA	Fenpropathrin	Green Tea	0.11 µg/g	0.64 µg/g	5 min	This work
CNPs-LFIA			0.017 µg/g	0.08 µg/g	5 min	This work

- Not mentioned.

Table S2 Cross-reactivity rate of FPT with main analogs (n=6).

Samples	AuNPs-LFIA (%)	CNPs-LFIA (%)
FPT	98.41	101.84
Cycloprothrin	4	4
Bifenthrin	4	4
Isocarbophos	4	4
Chlorpyrifos	4	4
Thiamethoxam	4	4
Carbofuran	4	4

Table S3. Recovery test for FPT using AuNPs-based LFIA and CNPs-based LFIA (n=6).

Method	Spiked Concentration (μg/g)	Mean Recovery (%)	Coefficient of Variation (%)
AuNPs-based LFIA	0.64	90.9	10.67
	1	97.8	11.75
	2	106.5	6.44
	4	87.4	8.76
CNPs-based LFIA	0.05	105.6	7.81
	0.1	89.7	12.84
	0.2	96.4	6.96
	0.4	92.7	9.72

Table S4. Detection of FPT in real green tea samples using AuNPs-based LFIA, CNPs-based LFIA, and GC-MS/MS (n=6).

Samples	AuNPs-LFIA (μg/g)	CNPs-LFIA (μg/g)	GC-MS/MS (μg/g)
Negative control	ND[a]	ND	ND
1	ND	ND	ND
2	ND	ND	ND
3	ND	ND	ND
4	ND	ND	ND
5	ND	ND	ND
6	ND	ND	ND
7	ND	ND	ND
8	ND	ND	ND
9	ND	ND	ND
10	ND	ND	ND
Negative control	ND	ND	ND
11	ND	ND	ND
12	ND	ND	ND
13	ND	ND	ND
14	ND	ND	ND
15	ND	ND	ND
16	ND	ND	ND
17	ND	ND	ND
18	ND	ND	ND
19	ND	ND	ND
20	ND	ND	ND

[a] means not detected.

References

(1) Xu, Z.; Wang, J.; Ye, Q.; Jiang, L.; Deng, H.; Liang, J.; Chen, R.; Huang, W.; Lei, H.; Xu, Z.; Luo, L., Highly selective monoclonal antibody-based fluorescence immunochromatographic assay for the detection of fenpropathrin in vegetable and fruit samples. *Anal. Chim. Acta* **2023,** 1246, 340898.

(2) Wang, X.; Huang, H.; Zhong, S.; Shentu, X.; Ye, Z.; Yu, X., Carboxymethyl chitosan–modified UiO-66 for the rapid detection of fenpropathrin in grains. *Int. J. Biol. Macromol.* **2024,** 265, 131032.

(3) Song, Y.; Jin, J.; Hu, L.; Hu, B.; Wang, M.; Guo, L.; Lv, X., Core-Shell-Shell upconversion nanomaterials applying for simultaneous immunofluorescent detection of fenpropathrin and procymidone. *Foods* **2023,** 12, 3445.

(4) Gao, J.; Zhang, T.; Fang, Y.; Zhao, Y.; Yang, M.; Zhao, L.; Li, Y.; Huang, J.; Zhu, G.; Guo, Y., On-site rapid detection of multiple pesticide residues in tea leaves by lateral flow immunoassay. *J. Pharm. Anal.* **2023,** 14, 276-283.

(5) Chen, X.; Liu, L.; Kuang, H.; Song, S.; Xu, C., A strip-based immunoassay for rapid determination of fenpropathrin. *Anal. Methods* **2013,** 5, 6234.
